# Effects of Dietary Yeast (*Saccharomyces cerevisia*) Supplementation in Practical Diets of Tilapia (*Oreochromis niloticus*)

**DOI:** 10.3390/ani2010016

**Published:** 2012-01-13

**Authors:** Rodrigo O. A. Ozório, Leandro Portz, Ricardo Borghesi, José E. P. Cyrino

**Affiliations:** 1 CIMAR/CIIMAR, Centro Interdisciplinar de Investigação Marinha e Ambiental, Universidade do Porto, 4050-123, Portugal; 2 Universidade Federal do Paraná, Palotina, Paraná, 85950-000, Brazil; E-Mail: lportz@ufpr.br; 3 Departamento de Zootecnia, Escola Superior de Agricultura Luiz de Queiroz, Setor Piscicultura, 13418-900, Piracicaba, São Paulo, Brazil; E-Mails: borghesi@cpap.embrapa.br (R.B); jepcyrin@esalq.usp.br (J.E.P.C)

**Keywords:** brewer’s yeast, *Saccharomyces cerevisiae*, growth performance, nutrient utilization, tilapia, *Oreochromis niloticus*

## Abstract

**Simple Summary:**

World communities are concerned about the increasing impact of the aquaculture activities on fisheries resources. Aquaculture sector uses 2–5 times more fishmeal to feed farmed species than what is supplied by the farmed product. Therefore, the reduction of fishmeal dependency may provide more economic and environmentally friendly aquaculture. By identifying alternative protein sources, the authors find that brewer’s yeast is a suitable raw material as fishmeal replacement in feed of tilapia. The 15% inclusion may promote growth without affecting the end-product quality.

**Abstract:**

A 51-day feeding trial was carried out to determine the effects of various dietary levels of brewer’s yeast, *Saccharomyces cerevisiae*, in the growth performance, body composition and nutrient utilization in Nile tilapia, *Oreochromis niloticus*, juveniles. Fish (7.6 ± 0.3 g) were stocked into eighteen 1,000-L tanks (100 fish per tank; n = 3) and fed to apparent satiation six isonitrogenous (27% crude protein) and isoenergetic (19 kJ/g) diets, formulated to contain different dried yeast levels (0%, 10%, 15%, 20%, 30% or 40% diet) in substitution to fishmeal. Body weight tripled at the end of the feeding trial for fish fed up to 20% dietary yeast incorporation. Daily growth coefficient (DGC, % body weight/day) decreased with increasing dietary yeast level (*P* < 0.0001). Voluntary feed intake (VFI, %BW/day) did not vary significantly with increasing yeast level. Fish fed 40% yeast showed significant reduction in protein efficiency rate, protein retention and nitrogen gain. Increasing levels of dietary yeast did not significantly affect protein or lipid digestibility. Dietary dried yeast was seemingly palatable to tilapia juveniles and was suitable up to 15% inclusion to promote growth and efficient diet utilization, without affecting body composition.

## 1. Introduction

Aquaculture systems currently use 2–5 times more fishmeal to feed farmed species than what is supplied by the farmed product [1]. The scarcity of high quality fishmeal and the wide gap in demand and supply for this resource are boosting its price and may eventually hamper further development of aquaculture [2,3]. Therefore, efforts have been made worldwide to find locally available protein sources to replace fishmeal from aquafeeds and relieve the pressure on fisheries resources.

A potential fishmeal surrogate, dried surplus yeast *Saccharomyces cerevisiae* from fermentation plants utilizing sugar cane, is commonly available in Brazil but its use in aquafeeds is still not fully optimized. Dried yeast has antinutritional-factors (e.g., nucleic acids), which if supplemented at high concentration may hamper the performances of monogastric animals including fish [4]. The use of brewer’s yeast at probiotic levels (up to 2%) has proven to have a positive effects on the performance and welfare in several fish species, such as African catfish *Clarias gariepinus* [5] and hybrid striped bass *Morone chrysops* × *M. saxatilis* [6]. However, in studies where brewer’s yeast was tested as protein source, *i.e.*, when high levels are required, the results are not entirely clear. Rainbow trout (*Oncorhynchus mykiss*) and tilapia (*Oreochromis niloticus*) fed with diets containing moderate to high yeast levels were reported to have reduced feed intake and growth, reduced liver glycogen and increased total liver lipids [7,8,9,10]. Conversely, the inclusion of 30–50% brewers yeast in the diet improved feed efficiency of European seabass [11] and Pacu, *Piaractus mesopotamicus* [12]. The aim of this study was to investigate the effects of partial and total fishmeal replacement by brewer’s yeast on growth, body composition, feed utilization and digestibility of juvenile tilapia. were checked in a daily basis. The trials were carried out at the Laboratory of Fish Nutrition of the College of Agriculture ‘Luiz de Queiroz’ (University of São Paulo, Brazil).

## 2. Experimental Section

### 2.1. Fish and Husbandry Conditions

Sex-reversed, full-siblings Nile tilapia (*Oreochromis niloticus*) juveniles (7.6 ± 0.3 g), with identical nutritional history, were randomly stocked and acclimatized to eighteen indoor 1,000-L tanks (100 fish pert tank) in a closed water recirculation system equipped with mechanical and biological filters, under a constant photoperiod (14 L:10 D). Water temperature (25.5 ± 1.4 °C), pH (7.9 ± 0.2), conductivity (2.7 ± 0.2 mS·cm^−1^), dissolved oxygen (DO; always > 6 mg·L^−1^) and total ammonia (<0.05 mg L^-1^)

### 2.2. Experimental Diets

Fish were hand-fed to apparent satiation, twice a day (09:00 and 17:00), with six isonitrogenous (27% protein) and isoenergetic (19 Kj·g^ −1 ^) diets, formulated with natural ingredients containing various dried yeast levels (0%, 10%, 15%, 20%, 30%, 40%) in substitution to fishmeal (Table 1(a)). The fishmeal replacement was carried out in such a way that all diets had approximately the same amino acid profile (Table 1(b)). The inclusion of purified lysine and methionine increased with the yeast level.

Dietary ingredients were homogeneously ground to 500 µm, thoroughly mixed, and humidified to 25–50%, depending on the diet formulation. Moist mixtures were cold-extruded through a 1.0 mm die mincer, dried overnight in a forced-air oven (45 °C), crumbled and sieved into 0.5–3.0 mm pellets. All diets were kept frozen (−20 °C) until they are distributed.

**Table 1:** (**a**) Feed formulation and nutrient composition of the experimental diets (% as-fed basis, unless otherwise stated). (**b**) Amino acid composition of the experimental diets (% as-fed basis).

( **a**)						
	Dietary yeast level (%)					
Ingredients	0	10	15	20	30	40
Fishmeal ^1^	25.0	18.0	16.0	13.0	8.0	0.0
Dried yeast ^2^	0.0	12.0	16.0	19.0	27.0	38.0
Soya meal ^3^	15.7	18.6	19.0	21.9	24.4	28.5
Starch	18.0	18.0	18.0	18.0	18.0	18.0
Soya oil	2.7	3.6	3.8	4.2	4.8	5.2
Wheat	29.5	20.0	17.1	13.6	6.9	0.0
Premix ^4^	0.35	0.35	0.35	0.35	0.35	0.35
Binder	2.5	2.5	2.5	2.5	2.5	2.5
Fosforin ^5^	0.0	1.5	1.0	1.5	1.5	1.9
Cellulose	4.7	3.9	4.6	4.4	4.8	3.6
L-lysine-HCL	0.6	0.7	0.6	0.7	0.8	0.8
DL-methionine	0.4	0.4	0.5	0.5	0.5	0.6
Cr_2_O_3_	0.5	0.5	0.5	0.5	0.5	0.5
	*Nutrient composition*					
Dry matter	97.0	96.9	95.6	96.6	96.3	95.8
Crude protein	28.3	27.1	27.4	27.1	27.2	26.3
Crude fat	9.8	10.3	10.3	11.1	11.4	11.9
Ash	9.8	10.2	9.7	9.5	9.0	8.5
Phosphorus	1.0	1.2	1.0	1.2	1.0	0.9
Gross energy (kJ·g^−1^)	18.7	18.5	18.7	18.8	18.9	18.8
(**b**)						
	*Indispensable amino acids*					
Arginine	1.6	1.5	1.4	1.5	1.4	1.4
Histidine	0.8	0.7	0.7	0.7	0.6	0.6
Isoleucine	0.9	0.9	0.9	1.0	1.0	1.0
Leucine	1.8	1.7	1.7	1.7	1.7	1.6
Lysine	2.0	2.0	1.9	2.0	2.0	1.9
Valine	1.1	1.1	1.1	1.1	1.1	1.1
Phenylalanine	1.0	1.0	1.0	1.0	1.0	0.9
Methionine	0.8	0.8	0.8	0.9	0.8	0.9
Threonine	1.0	1.0	1.0	1.0	1.0	1.0
	*Dispensable amino acids*					
Alanine	1.4	1.4	1.4	1.4	1.4	1.3
Aspartic acid	2.3	2.3	2.2	2.3	2.3	2.3
Cystine	0.3	0.3	0.3	0.3	0.3	0.4
Glycine	1.5	1.3	1.3	1.2	1.1	1.0
Glutamic acid	3.6	3.6	3.6	3.7	3.7	3.7
Serine	1.0	1.1	1.1	1.1	1.1	0.9
Tyrosine	0.7	0.7	0.7	0.7	0.7	0.8

^1^ Super Prime: dry matter, 90%; crude protein, 67%; lysine, 5.0%; methionine, 1.9%; threonine, 2.8%; triptophan, 0.67%; digestibility, 85%. ^2^ 92% dry matter; 39% crude protein; 0.3% crude lipids; 4% ash; 0.7% crude fiber; 3% lysine; 0.5% methionine, 2.2% threonine (“Roller Drum Drier Molasses Yeast”, ICC Brazil Ltda.). ^3^ Hi-PRO (Agroceres S/A): crude protein, 47.5%; lysine, 3.05%; methionine, 0.68%; threonine 1.95%; triptophan, 0.65%. ^4^ Contained per kg mixture: vitamin: A, 6,000,000 IU; B_1_, 5,000 mg; B_2_, 1,120 mg; B_3_, 30,000 mg; B_5_, 30,000 mg; B_6_, 8,000 mg; B_8_, 2,000 mg; B_9_, 3,000 mg; B_12_, 20,000 μg; C, 500 mg; D_3_, 2,250,000 IU; K_3_, 3,000 mg; E, 75,000 mg. Minerals: 150,000 ZnSO_4_; 60,000 MnSO_4_; 4,500 KI, FeSO_4_ 100,000 mg; 2,000 mg CoSO_4_; Na_2_SeO_3_ 400 mg. ^5^ Phosphorus concentrate

### 2.3. Sampling, Analytical Procedure and Measurements

Fish were sampled at the beginning (15 fish·tank^−1^) and at the end (50 fish·tank^−1^) of the trial, sacrificed by an overdose of benzocaine (0.2 g·L^−1^) and weighed to the nearest 0.1 g. A pool of fish sample from each tank was then ground and immediately freeze-dried and stored at −20 °C pending analyses.

Growth performances were measured as weight gain, feed conversion ratio (FCR), voluntary fed intake (VFI, g·kg^−1^·day^−1^), daily growth coefficient (DGC), protein efficiency ratio (PER), and nutrient retention and losses. Diets, faeces and carcass samples were submitted to proximate composition analysis. Dry matter (4 h, 105 °C) and ash (5 h, 550 °C) were determined on fresh matter basis. Total lipids (petroleum ether extraction, Soxhlet method, 40–60 °C), gross energy (Parr 1261 Oxygen Bomb Calorimeter, Parr, Moline, IL, USA), crude protein (macro-Kjeldahl; N × 6.25), crude fibre (Weende method), phosphorus (colorimetric method, after mineralization and acid hydrolysis) and the chromium content were determined on freeze-dried material according to Furukawa and Tsukahara [13]. Amino acid analysis was carried out in duplicate according to Ozório *et al*. [12].

The digestibility study was conducted using the settling column approach (Guelph system) for faeces collection as described by Cho *et al*. [14]. Fish were fed once per day with diets containing Cr_2_O_3_ as inert marker, and then transferred to 200-L conical-bottomed tanks, with continuous water flow (0.5 L·min^−1^; 25 ± 1 °C) and continuous aeration (DO > 6.0 mg·L^−1^). Faeces were collected into refrigerated containers for 15 hours after the afternoon meal during the last week of the feeding trial. Apparent digestibility coefficients (ADCs) of dietary protein and lipid were calculated by the formula ADC (%) = [1 – (F/D × DC/FC)] × 100; where F is the percent of nutrient in faeces, D is the percent of nutrient in diet, DC is the percent of chromic oxide in diet and FC is the percent of chromic oxide in faeces [15].

### 2.4. Statistical Analyses

Data were submitted to one-way ANOVA followed by Tukey’s multiple range test; normality assumptions were ensured through the Kolmogorov–Smirnov test; homogeneity of variance was established through Bartlett’s test [16]. Data were expressed as the mean ± SD of the replicates, each tank representing one experimental unit. Differences were considered significant if *P* was less than 0.05. All statistical analyses were performed using SAS software (SAS User’s Guide: Statistics, Version 9.0; SAS Institute, Cary, NC, USA).

## 3. Results and Discussion

Dried yeast is an ingredient that has been increasingly used in fish and shrimp feeds in countries with ethanol or other distillation plants. In the current study the partial fishmeal replacement by brewers yeast, *Saccharomyces cerevisiae*, have improved the nitrogen (N) gain and protein efficiency ratio in Nile tilapia (*Oreochromis niloticus*) when supplemented at a maximum of 10%, but had no significant effect on growth performance (Table 2). A linear depression on growth performance and nutrient retention were observed when fish were fed diets with more than 10% yeast (equivalent to ≥50% fishmeal substitution). Nevertheless, feed conversion ratio were statistically different from control group only in fish fed at 40% yeast, while voluntary feed intake did not differ among the dietary groups.

Nitrogen (N) gain decreased linearly from 515 to 315 mg·kg^−1^ ABW/day (*P* < 0.001), while protein digestibility remained unaltered (Table 3). Such combination of results may indicate an increase in mobilization of dietary protein for catabolic purposes and/or a reduction of intestinal absorptive ability when fish is fed increasing dietary yeast levels, both causing a decrease in the efficiency of dietary protein deposition.

Contrary to our findings, Oliva-Teles and Gonçalves [11] and Ozorio *et al.* [12] reported that yeast can replace 50% of fishmeal protein in seabass (*Dicentrarchus labrax*) and pacu diet, respectively, with no negative effects on the growth performances. In addition, Lara-Flores *et al*. [17] found that 40% dietary inclusion of yeast positively stimulated growth performance in tilapia.

**Table 2:** Effect of different dietary yeast levels on weight gain, feed efficiency and nutrient utilization in juvenile tilapia (initial weight = 7.6 ± 0.3 g) fed over 51 days.

	Dietary yeast level (%)						
*Performance*	0	10	15	20	30	40	ANOVA *P > F*
Final body weight (g)	28.8 ± 1.5 ^ab^	26.5 ± 1.6 ^ab^	24.4 ± 1.8 ^bc^	21.8 ± 0.2 ^cd^	19.7 ± 0.7 ^de^	15.6 ± 1.5 ^e^	0.0001
DGC ^1^	2.11 ± 0.12 ^a^	2.08 ± 0.14 ^a^	1.87 ± 0.22 ^ab^	1.64 ± 0.04 ^bc^	1.42 ± 0.09 ^c^	1.02 ± 0.17 ^d^	0.0001
FCR ^2^	1.5 ± 0.1 ^a^	1.5 ± 0.1 ^a^	1.5 ± 0.2 ^a^	1.7 ± 0.1 ^ab^	1.8 ± 0.1 ^ab^	2.1 ± 0.0 ^b^	0.0001
VFI ^3^	3.14 ± 0.09	3.26 ± 0.37	2.98 ± 0.17	3.05 ± 0.14	2.92 ± 0.03	2.63 ± 0.38	NS
PER ^4^	1.9 ± 0.3 ^a^	2.4 ± 0.2 ^b^	2.5 ± 0.12 ^b^	2.5 ± 0.4 ^b^	2.2 ± 0.2 ^ab^	2.1 ± 0.1 ^a^	0.04
N gain ^5^ (mg·kg^−1^ABW·day^−1^)	514.9 ± 12.4 ^a^	527.1 ± 35.4 ^a^	455.9 ± 55.8 ^ab^	424.8 ± 16.6 ^ab^	385.8 ± 24.3 ^bc^	312.4 ± 60.6 ^c^	0.0002
*Retention *^6^							
Dry matter	19.6 ± 0.9 ^a,b^	20.1 ± 1.3 ^a^	19 ± 3.4^ab^	17.6 ± 1.2 ^ab^	16.7 ± 0.9 ^ab^	15 ± 0.7 ^b^	0.02
Crude Protein	36.3 ± 1.9 ^a^	37.4 ± 2.2 ^a^	35.1 ± 3.4^a^	32.3 ± 2.5 ^ab^	30.4 ± 1.6 ^b^	28.2 ± 1.3 ^b^	0.02
Crude Lipid	64.4 ± 4.1 ^a^	62.5 ± 4.3 ^ab^	59.4 ± 9.4^ab^	56.4 ± 2.1 ^ab^	48 ± 7 ^b^	49.2 ± 2.3 ^b^	0.01
Gross Energy	25.7 ± 1.1	26.1 ± 3.7	25.7 ± 3.4	23.4 ± 1.7	22.7 ± 1.9	21 ± 0.8	NS

^1^ Daily Growth Coefficient: (% day^−1^): (FBW^1/3^ − IBW^1/3^)/54 days × 100. ^2^ Feed conversion ratio (g·g^−1^): wet weight gain / dry feed intake. ^3^ Voluntary feed intake (g·kg^−1^ ABW day^−1^): crude feed intake/ABW/trial duration, where ABW, average body weight: (IBW + FBW)/2/1,000 (kg). ^4^ Protein efficiency ratio (%): wet weight gain/crude protein intake. ^5^ Nitrogen gain: (final carcass nitrogen content − initial carcass nitrogen content)/ABW/54 days. ^6^ Nutrient retention (% intake): 100 × (nutrient gain/nutrient intake).

**Table 3:** Effect of different dietary yeast levels on whole-body composition and nutrient digestibility in juvenile tilapia (7.6 ± 0.3 g) fed over 51 days (% dry matter basis, unless otherwise stated).

		Dietary yeast level (%)							
	*Initial*	0	10	15	20	30		40	ANOVA *P > F*
Dry matter	22.87^a^	26.2 ± 0.3^c^	27.2 ± 0.5^c^	25.6 ± 0.7^bc^	26.2 ± 0.2^c^	26.3 ± 0.3^c^		26.4 ± 1.1^c^	0.0001
Crude protein	60.6^a^	54.3 ± 0.2^b^	52.7 ± 0.7^bc^	53.4 ± 0.7^b^	53.0 ± 0.2^b^	53.5 ± 0.5^b^		54.2 ± 0.8^b^	0.0001
Crude lipid	17.9^a^	25.3 ± 0.5^c^	25.6 ± 1.9^c^	24.3 ± 2.4^c^	25.9 ± 0.7^c^	24.4 ± 1.3^c^		23.5 ± 1.7^bc^	0.0001
Ash (DM)	16.4^a^	13.3 ± 0.2^c^	14.5 ± 1^bc^	14.6 ± 1.5^bc^	14.4 ± 1.3^bc^	15.3 ± 1.8^c^		15.6 ± 0.7^c^	0.0001
Gross energy (kJ·g^−1^)	14.5^a^	22.1 ± 0.3^b^	21.8 ± 1.9^b^	22.4 ± 0.4^b^	21.9 ± 0.7^b^	21.9 ± 0.7^b^		21.1 ± 0.6^b^	0.0001
*Apparent Digestibility (%)*									
Protein		82.3 ± 2.3	81.3 ± 0.2	78.6 ± 3.0	78.1 ± 2.1	79.4 ± 2.3	78.3 ± 1.6		NS
Lipid		87.3 ± 5.7	84.9 ± 3.1	87.2 ± 2.6	87.6 ± 2.2	89.7 ± 1.2	94.2 ± 1.6		NS

The reduced N gain obtained in the current study could be explained if the experimental diets had sub-optimum amino acid patterns, as lower N retention corresponds to higher N excretion and lower rates of protein growth. Since the amino acid levels did not vary significantly among the experimental diets (Table 1), it seems that the high dietary yeast level did not cause amino acid deficiencies.

The utilization of dried yeast at reduced levels may effectively improve growth [8,11,18] and non-specific immune responses [19,20,21,22] in a variety of fish species. As inasmuch, dried yeast is a source of nucleic acids and non-starch polysaccharides, including β-1,3 glucan, which in high concentrations may play a role of antinutritional factors. At high concentrations, such compounds are known to hamper nutrient digestion and/or absorption. In avian species, β-glucans may affect the absorption of nutrients, possibly by increasing gut viscosity [23], while high concentration in nucleic acids may affect nutrient metabolism in humans and most monogastric animals [4].

The carcass composition and the digestibility coefficients of the dietary nutrients are shown in Table 3. Carcass composition was not significantly affected by increasing levels of dietary yeast, with exception of ash content. Ash content was significantly higher in fish fed 30% and 40% yeast (*P* < 0.05) than the other dietary groups. The digestibility coefficients of protein (78–82%) and lipid (87–94%) were not significantly different with increasing dietary yeast (Table 3), suggesting that protein from yeast was well digested by tilapia. Our results are in agreement with Olvera-Novoa *et al.* [24] which observed similar digestibility values in tilapia fed with diet having similar yeast incorporation.

## 4. Conclusions

Overall, this study showed a linear decrease in growth performance and efficiency in nutrient utilization when fish were fed above 15% yeast, providing additional information for future studies interested in the optimization of a more economic and low-pollution non-fishmeal based diets.

## References

[1] Naylor R.L., Goldburg R.J., Primavera J.H., Kautsky N., Beveridge M.C.M., Clay J., Folke C., Lubchenco J., Mooney H., Troell M. (2000). Effect of aquaculture on world fish supplies. Nature.

[2] Tacon A.G.J., Dominy W.G. Overview of world aquaculture and aquafeed production. Proceedings of World Aquaculture 1999: the Annual International Conference and Exposition of the World Aquaculture Society.

[3] Siddhuraju P., Becker K. (2001). Preliminary nutritional evaluation of Mucuna seed meal (*Mucuna pruriens* var. *utilis*) in common carp (*Cyprinus carpio* L.): An assessment by growth performance and feed utilization. Aquaculture.

[4] Schulz E., Oslage H.J. (1976). Composition and nutritive value of single-cell protein SCP. Anim. Feed Sci. Technol..

[5] Essa M.A., Mabrouk H.A., Mohamed R.A., Michael F.R. (2011). Evaluating different additive levels of yeast, *Saccharomyces cerevisiae*, on the growth and production performances of a hybrid of two populations of Egyptian African catfish*, Clarias gariepinus*. Aquaculture.

[6] Li P., Gatlin D.M. (2003). Evaluation of brewers yeast (*Saccharomyces cerevisiae*) as a feed supplement for hybrid striped bass (*Morone chrysops* × *M. saxatilis*). Aquaculture.

[7] De la Huiguera M., Sanchez-Muniz F.J., Mataix F.J., Varela G. (1981). Nitrogen utilization by rainbow trout *Salmo gairdneri* fed on the yeast *Hansenula anomala*. Compar. Physiol. Biochem..

[8] Rumsey G.L., Kinsella J.E., Shetty K.J., Hughes S.G. (1991). Effect of high dietary concentrations of brewer’s dried yeast on growth performance and liver uricase in rainbow trout (*Oncorhynchus mykiss*). Anim. Feed Sci. Technol..

[9] Rumsey G.L., Winfree R.A., Hughes S.G. (1992). Nutritional-value of dietary nucleic-acids and purine-bases to rainbow-trout (*Oncorhynchus-mykiss*). Aquaculture.

[10] Baccarin A.E., Pezzato L.E., Urbinati E.C. (2000). Efeito da alimentação com levedura desidratada de álcool na glicemia e nos níveis de glicogênio e lipídeos totais hepáticos da tilápia do Nilo (*Oreochromis niloticus*). Boletim do Instituto de Pesca.

[11] Oliva-Teles A., Goncalves P. (2001). Partial replacement of fishmeal by brewers yeast (*Saccaromyces cerevisae*) in diets for sea bass (*Dicentrarchus labrax*) juveniles. Aquaculture.

[12] Ozório R.O.A., Turini B.G.S., Moro G., Oliveira L.S.T., Portz L., Cyrino J.E.P. (2010). Growth, nitrogen gain and indispensable amino acid retention of pacu (*Piaractus mesopotamicus*, Holmberg 1887) fed different brewers yeast (*Saccharomyces cerevisiae*) levels. Aquacult. Nutr..

[13] Furukawa A., Tsukahara H. (1966). On the acid digestion method for the determination of chromic oxide as an index substance in the study of digestibility of fish feed. Bull. Jpn. Soc. Sci. Fish..

[14] Cho C.Y., Slinger S.J., Bayley H.S. (1982). Bioenergetics of salmonid fishes: Energy intake, expenditure and productivity. Compend. Biochem. Phys..

[15] Cho C.Y., Kaushik S.J. (1990). Nutritional energetics in fish: Energy and protein utilization in rainbow trout (*Salmo gairdneri*). World Rev. Nutr. Diet..

[16] Zar J.H. (1996). Biostatistical Analysis.

[17] Lara-Flores M., Olvera-Novoa M.A., Guzmán-Méndez B.E., López-Madrid W. (2003). Use of bacteria *Streptococus faecium* and *Lactobacillus acidophilus*, and the yeast *Saccharomyces cerevisiae* as growth promoters in Nile tilapia *Oreochromis niloticus*. Aquaculture.

[18] Craig S.R., McLean E., Jacques K., Lyons P., Eds. (2006). Nutrigenomics in aquaculture research: A key in the Aquanomic revolution. Nutritional Biotechnology in the Food and Feed Industry.

[19] Paulsen S.M., Lunde H., Engstad R.E., Robertsen B. (2003). *In vivo* effects of beta-glucan and LPS on regulation of lysozyme activity and mRNA expression in Atlantic salmon (*Salmo salar* L.). Fish Shellfish Immunol..

[20] Rumsey G.L., Anderson D.P., Siwicki A.K. (1994). Dietary intake of immunostimulants by rainbow trout affects non-specific immunity and protection against furunculosis. Vet. Immunol. Immunopathol..

[21] Anderson D.P., Siwicki A.K., Rumsey G.L., Shariff M., Arthur J.R., Subasinghe R.P. (1995). Injection or immersion delivery of selected immunostimulants to trout demonstrate enhancement of non-specific defence mechanisms and protective immunity. Diseases in Asian Aquaculture: II Fish Health Section.

[22] Yoshida T., Kruger R., Inglis V. (1995). Augmentation of nonspecific protection in african catfish, *Clarias-gariepinus* (Burchell), by the long-term oral-administration of immunostimulants. J. Fish Dis..

[23] Bedford M.R., Classen H.L. (1992). Reduction of intestinal viscosity through manipulation of dietary rye and pentosanase concentration is effected through changes in the carbohydrate composition of the intestinal aqueous phase and results in improved growth rate and food conversion efficiency of broiler chicks. J. Nutr..

[24] Olvera-Novoa M.A., Martinez-Palacios C.A., Olivera-Castillo L. (2002). Utilization of torula yeast (*Candida utilis*) as a protein source in diets for tilapia (*Oreochromis mossambicus* Peters) fry. Aquacult. Nutr..

